# The role of regulatory T cells in antigen-induced arthritis: aggravation of arthritis after depletion and amelioration after transfer of CD4^+^CD25^+ ^T cells

**DOI:** 10.1186/ar1484

**Published:** 2005-01-11

**Authors:** Oliver Frey, Peter K Petrow, Mieczyslaw Gajda, Kerstin Siegmund, Jochen Huehn, Alexander Scheffold, Alf Hamann, Andreas Radbruch, Rolf Bräuer

**Affiliations:** 1 Institut fur Pathologie, Friedrich-Schiller-Universität, Jena, Germany; 2 Experimentelle Rheumatologie, Medizinische Klinik, Charité, Humboldt-Universität, c/o Deutsches Rheuma-Forschungszentrum, Berlin, Germany; 3 Deutsches Rheuma-Forschungszentrum, Berlin, Germany

**Keywords:** arthritis, regulatory T cells

## Abstract

It is now generally accepted that CD4^+^CD25^+ ^T_reg _cells play a major role in the prevention of autoimmunity and pathological immune responses. Their involvement in the pathogenesis of chronic arthritis is controversial, however, and so we examined their role in experimental antigen-induced arthritis in mice. Depletion of CD25-expressing cells in immunized animals before arthritis induction led to increased cellular and humoral immune responses to the inducing antigen (methylated bovine serum albumin; mBSA) and autoantigens, and to an exacerbation of arthritis, as indicated by clinical (knee joint swelling) and histological scores. Transfer of CD4^+^CD25^+ ^cells into immunized mice at the time of induction of antigen-induced arthritis decreased the severity of disease but was not able to cure established arthritis. No significant changes in mBSA-specific immune responses were detected. *In vivo *migration studies showed a preferential accumulation of CD4^+^CD25^+ ^cells in the inflamed joint as compared with CD4^+^CD25^- ^cells. These data imply a significant role for CD4^+^CD25^+ ^T_reg _cells in the control of chronic arthritis. However, transferred T_reg _cells appear to be unable to counteract established acute or chronic inflammation. This is of considerable importance for the timing of T_reg _cell transfer in potential therapeutic applications.

## Introduction

Rheumatoid arthritis (RA) is the most common autoimmune disease in humans, affecting 1% of the population in western countries. Histologically, RA is characterized by hyperplasia and infiltration of the synovial membrane with mononuclear cells, development of an aggressive tissue called pannus and secretion of proteases, which are responsible for the destruction of articular cartilage and adjacent bone. It is well established that macrophages and synovial fibroblasts are effector cells of joint destruction, and it is presumed that autoreactive CD4^+ ^T cells are involved in their activation [1]. There is now a large body of evidence that, in rodents, regulatory T cells (T_reg_) actively control the activation of autoreactive T cells and thus maintain immunological self-tolerance. Apart from adaptive T_reg _cells, which can be induced by antigen-specific stimulation of conventional peripheral T cells under tolerogenic conditions (for review [2]), there is no doubt that naturally occurring T_reg _cells exist in healthy mice as well as in humans and rats, and these are characterized by constitutive expression of CD25 [3-5]. Absence of these cells *in vivo *results in a multi-organ autoimmune syndrome [3,6]. These CD4^+^CD25^+ ^T_reg _cells leave the thymus as committed 'professional' suppressor T cells [7-9], proliferate in the periphery, and acquire an effector/memory-like phenotype [10]. In unmanipulated mice, T_reg _cells can also be found in the CD25^- ^compartment, based on the expression of the integrin α_E_β_7 _[10,11], possibly reflecting differences in developmental stages of these cells.

The exact role played by naturally occurring CD4^+^CD25^+ ^T_reg _cells in the pathogenesis of arthritis remains controversial. Arthritis is part of the autoimmune syndrome induced by transfer of CD25-depleted splenocytes into lymphopenic hosts [3], and CD4^+^CD25^+ ^cells are protective in collagen-induced arthritis [12]. However, Bardos and coworkers [13] ruled out a role for naturally occurring CD4^+^CD25^+ ^T_reg _cells in proteoglycan-induced arthritis.

To clarify this issue, we used the antigen-induced arthritis (AIA) model. AIA is a Tcell-dependent experimental arthritis that is induced by intra-articular injection of antigen (methylated bovine serum albumin [mBSA]) into knee joints of preimmunized mice [14,15]. This results in an acute inflammatory reaction, which is characterized by exudation of neutrophils and fibrin, which later proceeds to a chronic arthritis with synovial hyperplasia, infiltration of mononuclear cells, and cartilage and bone destruction – histopathological changes similar to those that occur in RA. Autoimmune responses against cartilage constituents such as collagen types I and II and proteoglycans are involved in rendering the disease chronic [16,17]. Beyond the 100% incidence of arthritis, another major advantage of the AIA model is that the time point of induction of arthritis is known, allowing manipulation of CD4^+^CD25^+ ^T_reg _cell number *in vivo *at defined stages in the disease. Using depletion of CD25-expressing cells or transfer of CD4^+^CD25^+ ^cells, in the present study we demonstrated that T_reg _cells modulate the onset of AIA but are ineffective at later stages, calling into question their value as a new therapeutic approach to established chronic arthritis.

## Methods

### Animals, arthritis induction and assessment

For all animal experiments, female C57Bl/6 mice (Charles River, Sulzfeld, Germany; age range 6–10 weeks) were used. Animals were kept under standard conditions, fed a standard diet and given free access to water. All animal studies were approved by the government commission for animal protection.

At 21 and 14 days before arthritis induction, mice were subcutaneously injected with 100 μg mBSA (Sigma, Deisenhofen, Germany), emulsified in complete Freund's adjuvant (Sigma) supplemented to 2 mg/ml heat-killed *Mycobacterium tuberculosis *(strain H37RA; Becton Dickinson [BD], Heidelberg, Germany). Simultaneously, mice received 5 × 10^8 ^heat-inactivated *Bordetella pertussis *(Chiron-Behring, Marburg, Germany) intraperitoneally. Arthritis was induced by intra-articular injection of 100 μg mBSA in 25 μl phosphate-buffered saline (PBS) into the right knee joint cavity.

Arthritis severity was monitored by measurement of lateral joint diameter using a vernier caliper (Oditest, Kroeplin Längenmesstechnik, Schlüchtern, Germany). Histological severity of arthritis was scored in a blinded manner by two investigators (PKP and MG) in frontal knee joint sections, stained with haematoxylin and eosin and prepared as described previously [14]. Briefly, at least four sections per knee joint were semiquantitatively examined on a 0–3 point scale for each of the following: extent of synovial hyperplasia, mononuclear infiltration, cartilage destruction and pannus formation.

### Antibodies and reagents

The following antibodies were grown and purified from the culture supernatants in our laboratory: anti-CD25 (PC61), anti-CD3 (145 2C11), anti-CD4-FITC and FITC-labelled anti-CD4-F(ab) (GK1.5), anti-CD8 (TIB105), anti-CD28 (37.51) and anti-Mac-1 (M1/70). The following antibodies and secondary reagents were purchased from BD Pharmingen (Heidelberg, Germany): PE-Cy5-labelled anti-CD4 (H129.9), biotinylated anti-α_E_β_7 _(M290), biotinylated anti-CD25 (7D4), allophycocyanine or FITC-conjugated anti-CD25 (PC61), streptavidin-allophycocyanine and streptavidin-PE, and matched antibody pairs for ELISPOT analysis of IFN-γ (R4-6A2 and biotinylated XMG1.2) and IL-4 (BVD4 1D11 and biotinylated BVD6-24G2) production.

### *In vivo *depletion

Mice were injected with 0.5 mg purified anti-CD25 antibody (PC61) 4 and 2 days before intra-articular antigen injection. Polyclonal rat IgG, purified from normal rat serum, was used as control. The degree of depletion was determined by fluorescence-activated cell sorting, using a non-cross-reactive biotin-labelled anti-CD25, FITC-labelled anti-CD4 and streptavidin-conjugated allophycocyanine. Measurement was performed using FACSCalibur^® ^(BD) and data were analyzed using WinMDI .

### Preparation, pre-activation and transfer of regulatory T cells

Pooled spleen and lymph node cells from naive C57Bl/6 donors or, if indicated, from immunized mice were incubated with anti-CD4-FITC (clone GK1.5) and anti-CD25-biotin (clone 7D4; BD). CD4^+ ^T cells were isolated using an anti-FITC-Multisort-Kit (Miltenyi Biotech, Bergisch-Gladbach, Germany) in accordance with the manufacturer's instructions. CD4^+ ^T cells were sorted into CD25^- ^and CD25^+ ^cells using anti-biotin MicroBeads (Miltenyi Biotech). Purity was greater than 92% for CD4^+^CD25^- ^and greater than 80% for CD4^+^CD25^+ ^cells.

CD25-expressing and α_E_β_7_-expressing subsets were sorted by FACS. Briefly, pooled spleen and lymph node cells from naive mice were stained with anti-CD25-FITC, anti-α_E_β_7_-biotin and streptavidin-PE. The stained cells were enriched with anti-FITC and anti-PE MicroBeads, using the AutoMACS separation unit (Miltenyi Biotech). Thereafter, the cells were sorted into subsets according to their expression of CD25 or α_E_β_7 _using a FACSDiVa cell sorter (BD). The purity was 90–95%, as determined by FACS.

For activation, cells were cultured for 24–72 hours in the presence of plate-bound anti-CD3 (3 μg/ml), anti-CD28 (10 μg/ml) and rhIL-2 (100 U/ml; Chiron, Ratingen, Germany) in RPMI 1640 containing 10% fetal calf serum (FCS; Gibco, Karlsruhe, Germany). Thereafter, cells were washed with PBS and transferred intravenously via lateral tail vein into mice at the time point of AIA induction or at later time points when indicated.

### Delayed-type hypersensitivity reaction

Seven days after arthritis induction, mice were challenged by intradermal injection into their ears of 5 μg mBSA in 10 μl PBS. Ear thickness was measured before injection and 24 and 48 hours later using a vernier caliper (Kroeplin).

### Proliferation assay and ELISPOT analysis

Single cell suspension from spleens and lymph nodes (inguinal, popliteal, axillary) were cultured at a density of 1 × 10^6^/ml in RPMI 1640, containing 10% FCS, 2 mmol/l L-glutamine, 10 mmol/l Hepes, 1 mmol/l sodium pyruvate, 0.5 μmol/l 2-mercaptoethanol and antibiotics (100 U/ml penicillin, 0.1 mg/ml streptomycin; all from Gibco) in the presence of medium alone or 25 μg/ml mBSA for 72 hours in 96-well tissue culture plates (Greiner Bio One, Nürtingen, Germany). Cells were pulsed with 0.5 μCi [^3^H]thymidine (Amersham-Buchler, Braunschweig, Germany) for the last 18 hours of culture. Thereafter, cells were harvested onto 96-well glass fibre filters (Packard Bioscience, Groningen, The Netherlands), and [^3^H]thymidine incorporation was measured with a scintillation counter (Top-Count; Packard Bioscience).

For ELISPOT analysis, PVDF-membrane 96-well microplates (Millipore, Eschborn, Germany) were coated overnight at 4°C with the primary antibody diluted in sterile PBS. After washing, plates were blocked for 2 hours with RPMI 1640 containing 10% FCS. Thereafter 2 × 10^5 ^(IL-2 and IFN-γ) or 1 × 10^6 ^(IL-4) cells were cultured in duplicate wells for 24 (IL-2 and IFN-γ) or 48 hours (IL-4). After washing again plates were incubated overnight at 4°C with the secondary antibody diluted in PBS/1% BSA. Extravidin–alkaline phosphatase conjugate (1:30,000 in PBS/1% BSA) and BCIP/NBT solution (bromochloroindolyle phophate/nitroblue tetrazolium; both from Sigma) were used for spot development. The number of spots was quantified using a KS-ELISPOT-Reader (Carl Zeiss, Oberkochen, Germany).

### Determination of serum IgG by ELISA

Microplates (96-well; Greiner Bio One) were coated with antigen (0.125 μg/ml mBSA), collagen type I (from rat tail tendon) and type II (10 μg/ml), and proteoglycans (10 μg/ml both from bovine cartilage) and left overnight, as described previously [14]. After washing, plates were incubated with serially diluted serum samples and the amount of bound IgG was determined using anti-mouse IgG-peroxidase conjugate (ICN, Eschwege, Germany) and ortho-phenylendiamine (Sigma) as substrate. Extinction was measured at 492 nm against 620 nm with an ELISA reader (Tecan, Crailsheim, Germany).

### Cell transfer for *in vivo *homing assay

For *in vivo *homing assay, cells were sorted with a modified protocol and labelled with ^111^indium, as described elsewhere [10]. Briefly, CD4^+ ^cells were enriched by negative selection. Enriched CD4^+ ^T cells were stained with FITC-conjugated anti-CD4-F(ab) and anti-CD25-allophycocyanine and sorted into CD4^+^CD25^+ ^or CD4^+^CD25^- ^cells by FACS (BD). Cells were labelled with ^111^In (Indiumoxin; Amersham-Buchler) for 20 min at room temperature; 1 × 10^6 ^labeled cells were injected intravenously, and 24 hours later mice were killed and the distribution of radioactivity in various organs and the rest of the body was measured in a γ-counter (Wallac Counter, Turku, Finnland).

Alternatively, a proportion of these cells was labelled with 5,6-carboxyfluorescein diacetate succinimidyl ester (CFSE) by incubation with 5 μmol/l CFSE (Molecular Probes, Leiden, The Netherlands) in RPMI 1640 for 5 min at room temperature. After washing, 1 × 10^6 ^cells were injected intravenously. Twenty-four hours later single cell suspensions were prepared from the draining and nondraining peripheral and mesenteric lymph nodes, the spleen and the peripheral blood, and stained with anti-CD4 and analyzed by FACS. Dead cells were excluded using propidiumiodide.

### Statistical analysis

Data are expressed as mean ± standard error of mean, unless otherwise indicated. Experimental groups were tested for statistically significant differences with the Mann–Whitney U-test using SPSS 10.0 (SPSS Inc, Chicago, IL, USA).

## Results

### Depletion of CD25-expressing cells exacerbates antigen-induced arthritis

**Figure 1:**
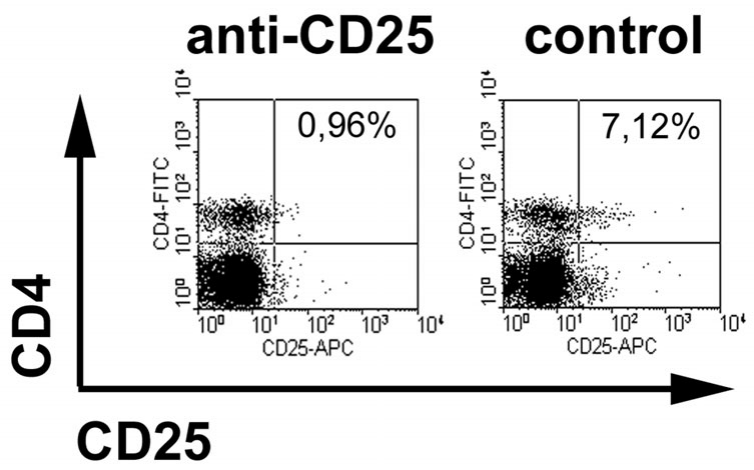
Depletion of CD25-expressing cells by anti-CD25 treatment. Mice immunized with methylated bovine serum albumin (mBSA) were injected intraperitoneally with 0.5 mg PC61 (anti-CD25) or rat IgG as control 4 and 2 days before arthritis induction. Representative example for flow-cytometric assessment of depletion in spleen cells, using a non-cross-reactive anti-CD25 antibody (7D4) at the time of arthritis induction (day 0).

Mice were injected intraperitoneally with 0.5 mg anti-CD25 (PC61) 4 and 2 days before induction of arthritis (i.e. 19 and 17 days after first immunization). Depletion of CD25-expressing cells was confirmed using FACS at the time of AIA induction (day 0) using an antibody that recognizes a different epitope on the CD25 molecule. In the PC61-treated group there was a 70.9 ± 11.4% (*n *= 3) reduction in CD4^+^CD25^+ ^cells in the spleens as compared with control mice injected with rat IgG (Fig. 1). Of note, the anti-CD25 treatment almost completely depleted cells with high expression of CD25, which are considered T_reg _cells, in contrast to CD4^+ ^T cells with low or intermediate levels of CD25 expression.

**Figure 2:**
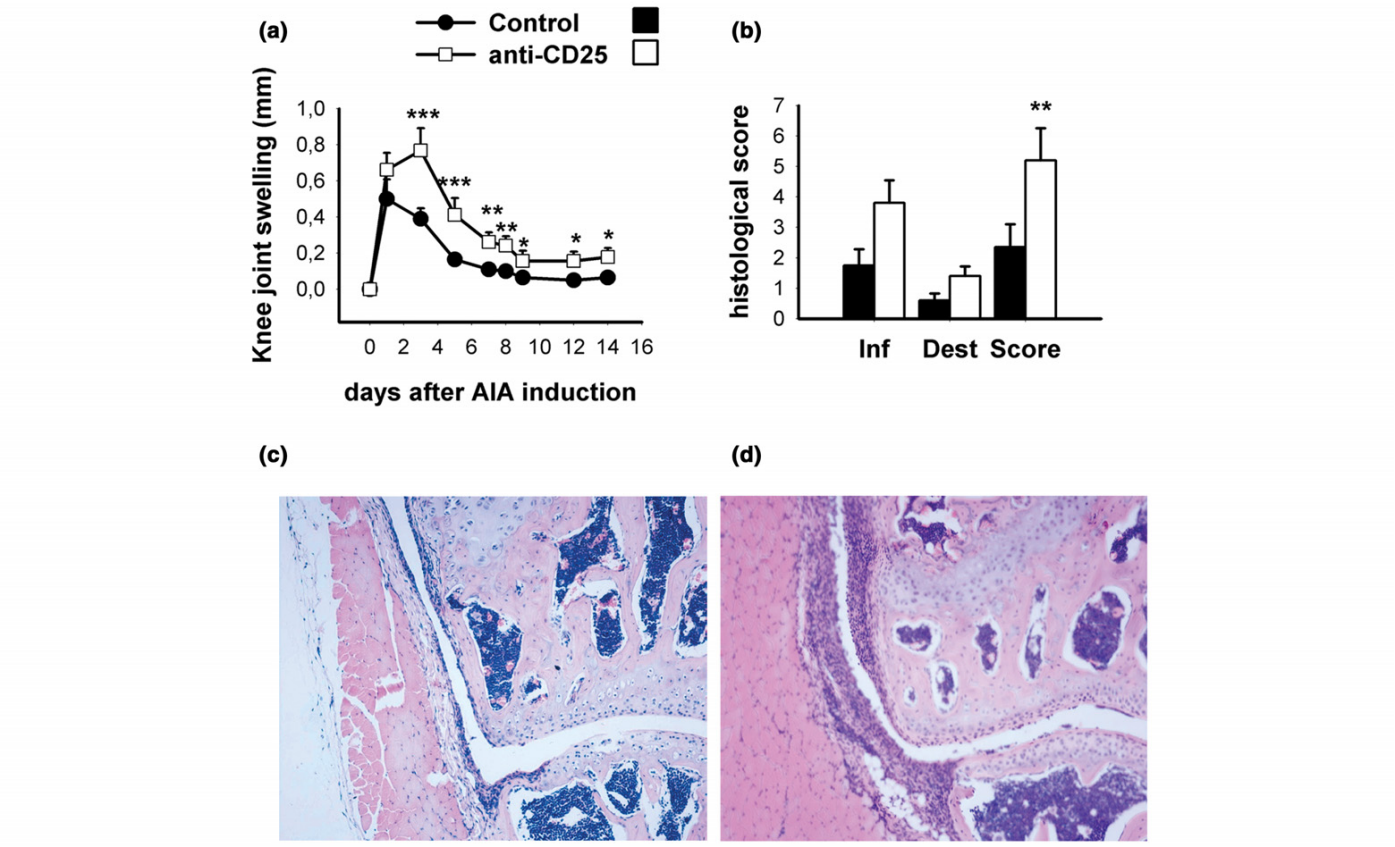
Clinical and histological severity of antigen-induced arthritis (AIA) in CD25-depleted mice. **(a) **Knee joint swelling (difference in mediolateral joint diameters of arthritic minus nonarthritic knee joints) during the time course of arthritis was higher in CD25-depleted mice. **(b) **Haematoxylin and eosin stained frontal knee joint sections were scored on a 0–3 point scale at day 14 of AIA for each of the following: severity of synovial hyperplasia, cellular infiltration, cartilage destruction and pannus formation. A score for inflammatory changes (Inf) was calculated by adding the points for synovial hyperplasia and infiltration, and for joint destruction (Dest) by adding the points for cartilage damage and pannus formation. Total arthritis score (Score) was calculated by adding scores for inflammatory changes and joint destruction, giving a maximal AIA score of 12 points. Representative photomicrographs of **(c) **a control (rat IgG-injected) and **(d) **a knee joint from an anti-CD25-treated mouse. Ten animals were included in each group in two independent experiments. *P < 0.05, **P < 0.01, ***P < 0.001, versus control.

After intra-articular antigen injection, knee joint swelling of the CD25-depleted mice was significantly greater from day 3 onward than in the control group injected with rat IgG (Fig. 2a). Histological examination of knee joint sections 14 days after AIA induction revealed increased hyperplasia and infiltration of the synovial membrane, as well as increased articular damage in those animals (Fig. 2b–d). In summary, this indicates a marked exacerbation of AIA by depletion of CD25-expressing cells.

### Increased cellular and humoral immune responsiveness in CD25-depleted mice

**Figure 3:**
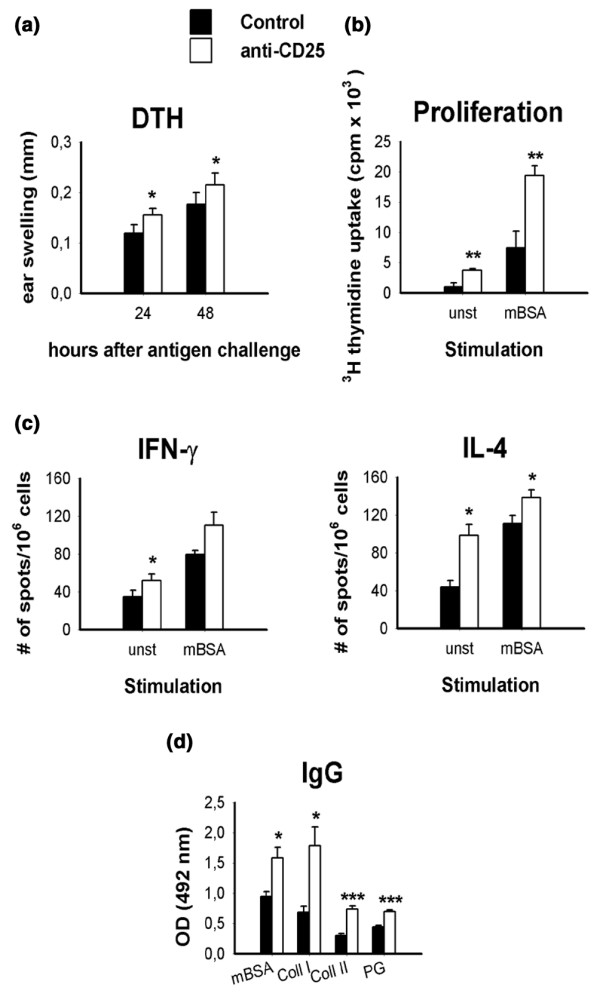
Analysis of *in vivo *and *ex vivo *immune responses in CD25-depleted mice. **(a) ***In vivo *delayed-type hypersensitivity (DTH) response against methylated bovine serum albumin (mBSA) as a marker for cellular immune response was measured as the increase in ear thickness after intradermal antigen challenge on day 7 of antigen-induced arthritis (AIA). **(b) **Proliferation, measured as [^3^H]thymidine incorporation of unstimulated (unst) or mBSA-stimulated (mBSA) draining lymph node cells at day 14 of AIA. **(c) **Cytokine production was measured with ELISPOT. **(d) **Serum levels of IgG against mBSA, collagen type I, collagen type II and cartilage proteoglycans were measured using ELISA after 14 days of AIA. Proliferation, DTH reaction and serum IgG titres were tested in 10 animals per group; cytokine production was measured in six animals per group. Data are from one out of two similar experiments. *P < 0.05, **P < 0.01, ***P < 0.001, versus control.

To assess how *in vivo *cellular immune responses against mBSA are influenced by depletion of CD25-expressing cells, delayed-type hypersensitivity (DTH) reaction against the same antigen was tested by intradermal injection of mBSA into the ears of mice at day 7 after induction of AIA. Anti-CD25 treated mice mounted a significantly stronger DTH response than did rat IgG-treated controls (Fig. 3a).

For analysis of the cellular immune responses *ex vivo*, draining lymph node cells of arthritic animals were harvested 14 days after AIA induction, restimulated with mBSA, and analyzed for proliferative response and cytokine production. As expected from the increased DTH reaction, the proliferative response to mBSA was significantly increased in cells from CD25-depleted mice as compared with that in rat IgG-treated controls (Fig. 3b). Importantly, even without antigenic stimulation the lymph node cells from CD25-depleted mice proliferated fourfold as much as cells from mice treated with control IgG. These data imply that a substantial proportion of the T-cell compartment is still activated 14 days after intra-articular antigen challenge in the absence of T_reg _cells.

Compatible with these findings is that the production of cytokines in response to mBSA was greater in CD25-depleted mice. Importantly, both T-helper-1 (IFN-γ) and T-helper-2 (IL-4) responses were aggravated by depletion of T_reg _cells, indicating that both types of response are subject to suppression by T_reg _cells (Fig. 3c). Again, cytokine secretion from T_reg_-depleted animals was increased even without antigenic stimulus. In accordance with this, serum levels of IgG directed against mBSA as well as levels of the cartilage-specific autoantigens collagen type I, collagen type II and proteoglycans, were found to be increased in CD25-depleted mice (Fig. 3d).

Taken together, these data clearly demonstrate that CD4^+^CD25^+ ^T_reg _cells regulate the severity of arthritis by limiting the cellular and humoral immune responses against the inducing antigen mBSA as well as some arthritis-related autoantigens.

### Transfer of CD4^+^CD25^+ ^cells

**Figure 4:**
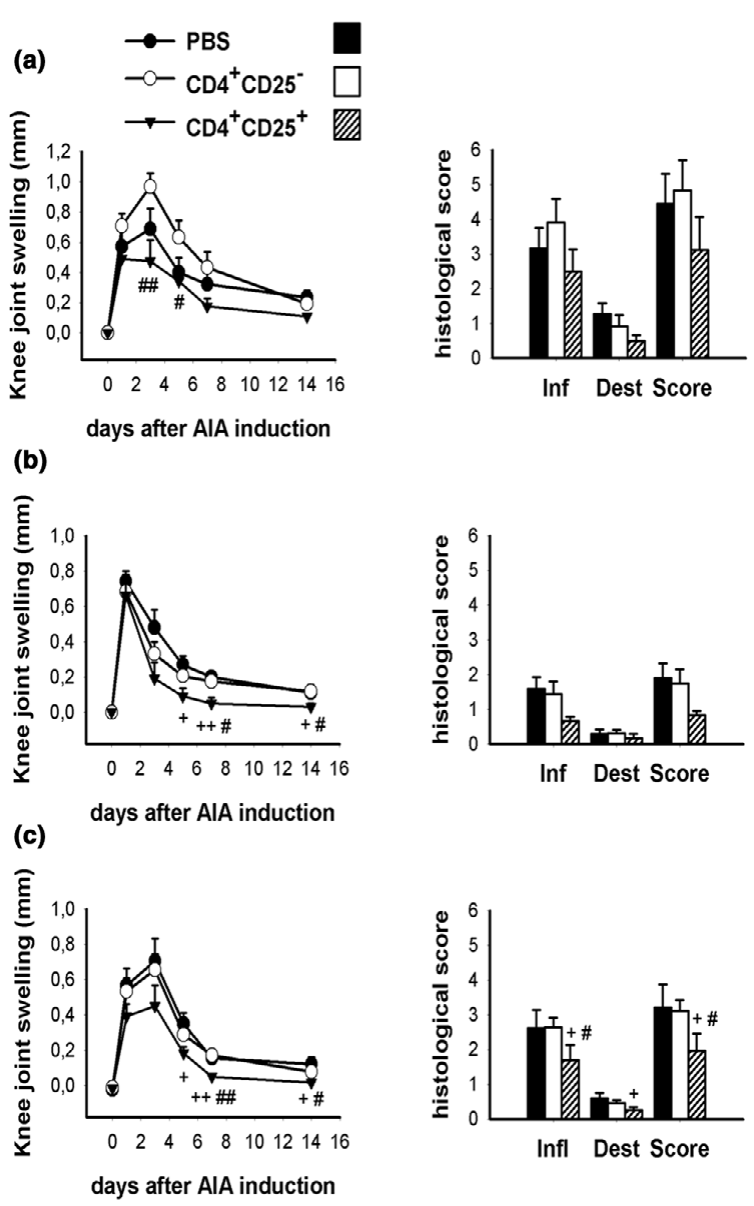
Modulation of antigen-induced arthritis (AIA) by transfer of regulatory T cells (T_reg _cells). Amelioration of clinical and histological severity of AIA by transfer of 2 × 10^6 ^CD4^+^CD25^+ ^cells freshly isolated from **(a) **naive or **(b) **immunized mice at the time of AIA induction (day 0; *n *= 6 per group). **(c) **Transfer of 1 × 10^6 ^*in vitro *pre-activated cells at the time of AIA induction (*n *= 6). ^#^*P *< 0.05, ^##^*P *< 0.01 for CD4^+^CD25^+ ^versus CD4^+^CD25^-^; ^+^*P *< 0.05, ^++^*P *< 0.01 for CD4^+^CD25^+ ^versus phosphate-buffered saline. Dest, joint destruction; Inf, inflammatory changes; Score; total arthritis score

To further characterize the suppressive potential of CD4^+^CD25^+ ^T_reg _cells, we performed cell transfer studies. In a first set of experiments we transferred T_reg _cells freshly isolated from naive (Fig. 4a) or mBSA/CFA immunized (Fig. 4b) mice into mBSA-immunized recipients at the time of intra-articular antigen challenge (day 0). With this protocol, a slight decrease in the severity of clinical arthritis (knee joint swelling) could be induced. Accordingly, the histological severity of AIA was also found to be reduced, albeit not statistically significantly (Fig. 4a, b).

It is known that T_reg _cells must be activated via their T-cell receptor to exert their suppressive function. Because we were unable to use antigen-specific (i.e. T-cell receptor transgenic) T_reg _cells, we opted to pre-activate the CD4^+^CD25^+ ^cells by *in vitro *culture in the presence of anti-CD3, anti-CD28 and IL-2 in order to increase their suppressive potential. Transfer of 1 × 10^6 ^pre-activated cells significantly suppressed both knee joint swelling and histological arthritis score (Fig. 4c). This effective suppression of AIA development was a consistent finding in different experiments, even with the use of lower cell numbers (for instance 2 × 10^5 ^cells; data not shown).

**Figure 5:**
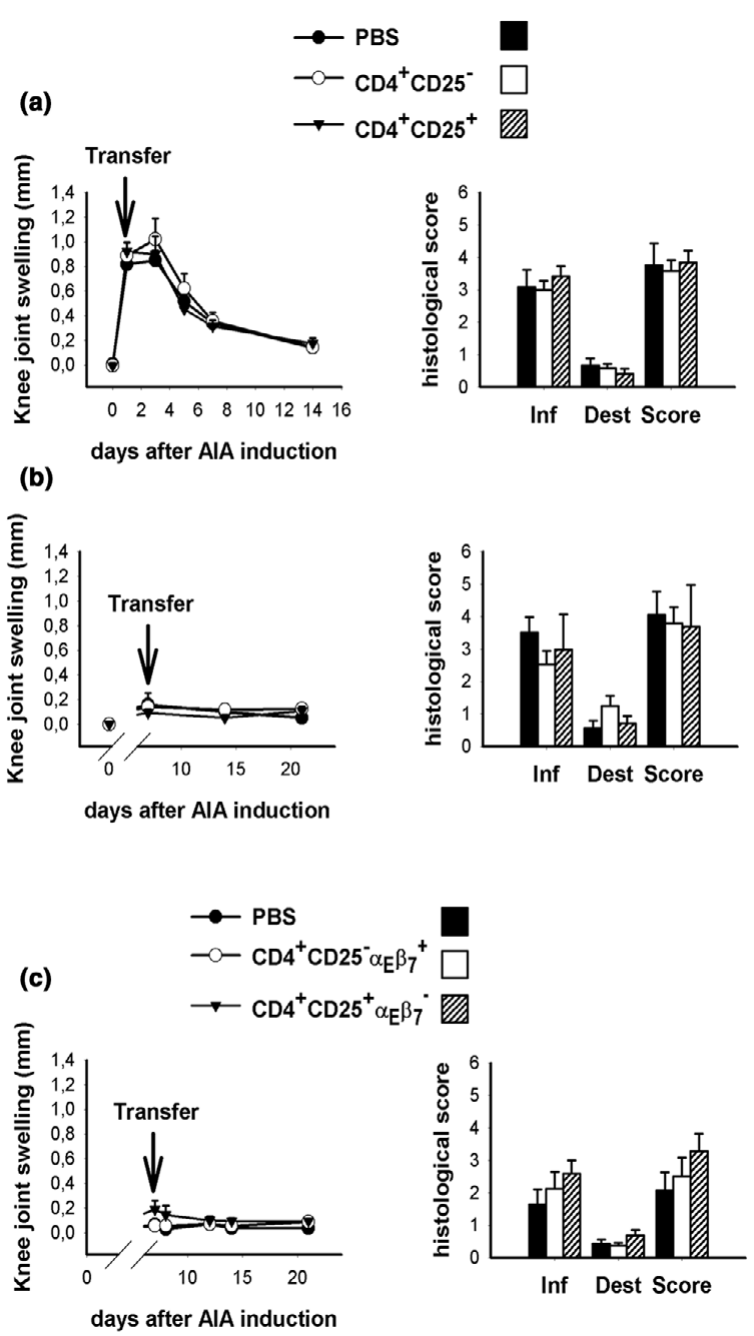
Transfer of regulatory T cells (T_reg _cells) cannot cure established arthritis. Pre-activated CD4^+^CD25^+ ^cells (1 × 10^6^) were transferred on **(a) **day 1 or **(b) **day 7 of antigen-induced arthritis (AIA). Arthritis severity was monitored by measurement of knee joint swelling and by histological assessment 14 days after cell transfer (*n *= 6–7 per group). **(c) **Also, 1 × 10^6 ^pre-activated α_E_β_7_-expressing T_reg _cells have no curative effect in AIA (*n *= 8 per group).

In the next step, we attempted to cure established arthritis by transfer of T_reg _cells. Surprisingly, 1 × 10^6 ^pre-activated CD4^+^CD25^+ ^cells had no influence on either knee joint swelling or histological arthritis score when transferred at day 1 (Fig. 5a) or day 7 (Fig. 5b) after induction of arthritis. Also, the transfer of 1 × 10^6 ^pre-activated α_E_β_7_-expressing T_reg _cells, which are highly effective in preventing AIA [10], had no effect on disease at this time point (Fig. 5c).

Taken together, our data demonstrate that T_reg _cells can inhibit arthritis development when transferred at the time of arthritis induction. However, we were unable to demonstrate any therapeutic effect of T_reg _cell transfer (in numbers that are effective in prevention) when performed after disease onset.

### Transferred CD4^+^CD25^+ ^T_reg _cells do not suppress humoral or cellular immune responses

**Figure 6:**
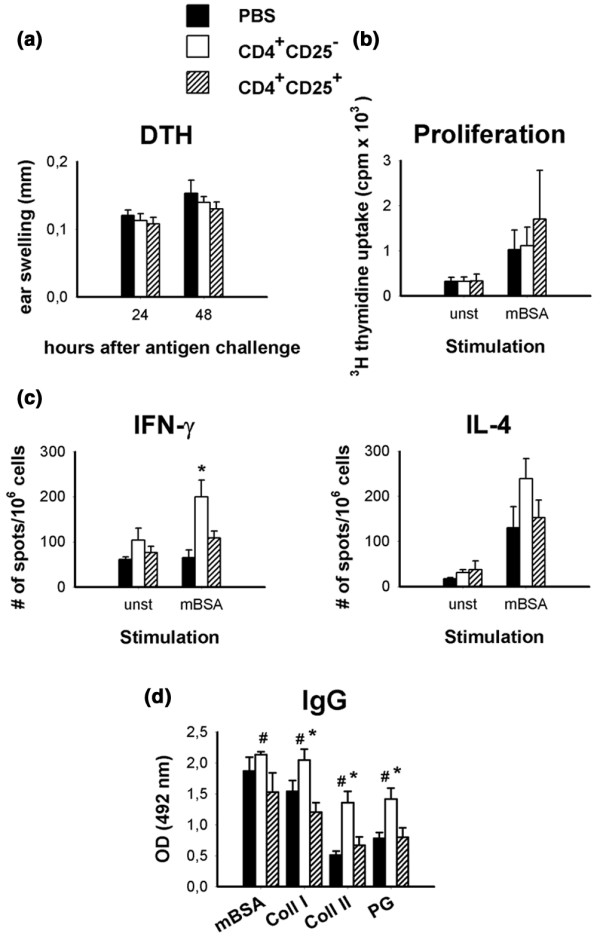
There is no suppression of cellular or humoral methylated bovine serum albumin (mBSA)-specific immunity with transfer of T_reg _cells. Pre-activated CD4^+^CD25^+ ^cells (1 × 10^6^) were transferred at the time of antigen-induced arthritis (AIA) induction. **(a) **Delayed-type hypersensitivity (DTH) reactivity against mBSA *in vivo *was tested 7 days later by an intradermal antigen challenge into the ears. **(b) **Antigen-specific proliferation ([^3^H]thymidine incorporation) and **(c) **cytokine production (ELISPOT) of draining lymph node cells was measured 14 days after AIA induction. **(d) **Serum levels of IgG against mBSA, collagen type I, collagen type II and cartilage proteoglycans were measured with ELISA after 14 days of AIA. Proliferation, DTH reaction, cytokine production, and serum IgG titres were tested in six animals per group. ^#^*P *< 0.05 for CD4^+^CD25^+ ^versus CD4^+^CD25^-^; *P < 0.05 for CD4^+^CD25^- ^versus phosphate-buffered saline.

Because CD25-depletion caused a substantial increase in both cellular and humoral immunoreactivity against mBSA, we examined whether transfer of CD4^+^CD25^+ ^T_reg _cells can suppress these responses. Neither DTH reactivity against mBSA (analyzed 7 days after AIA induction; Fig. 6a) nor mBSA-induced proliferation (Fig. 6b) and cytokine production by draining lymph node cells (Fig. 6c) at day 14 after induction of AIA were found to be suppressed in the recipients of 1 × 10^6 ^pre-activated CD4^+^CD25^+ ^cells. Thus, transfer of T_reg _cells into immunized animals does not eliminate or induce functional modification to the previously primed mBSA-specific immune response. In contrast, transfer of CD4^+^CD25^- ^cells did significantly enhance the proliferation as well as the cytokine production in the recipients. Accordingly, serum levels of IgG directed against mBSA and the cartilage-specific autoantigens collagen type I and type II, and proteoglycans were also not significantly diminished in T_reg _cell recipients compared with the saline-treated control group. Recipients of CD4^+^CD25^- ^cells had higher levels of IgGs (Fig. 6d).

### Homing properties of CD4^+^CD25^+ ^T_reg _cells

Because the mechanism of suppression of T_reg _cells *in vitro *is cell contact dependent, localization of cells might be important for their regulatory activity. Therefore, we investigated the migration behaviour of CD4^+^CD25^+ ^and CD4^+^CD25^- ^cells *in vivo*. For these experiments CD4^+ ^cells were enriched by negative selection and sorted by FACS into CD4^+^CD25^+ ^and CD4^+^CD25^- ^populations with preferential use of F(ab)-fragments or antibodies, which do not interfere with migration *in vivo*. Cells were labelled with ^111^In and injected intravenously into AIA mice 7 days after induction of arthritis. After 24 hours radioactivity was measured in different organs.

**Figure 7:**
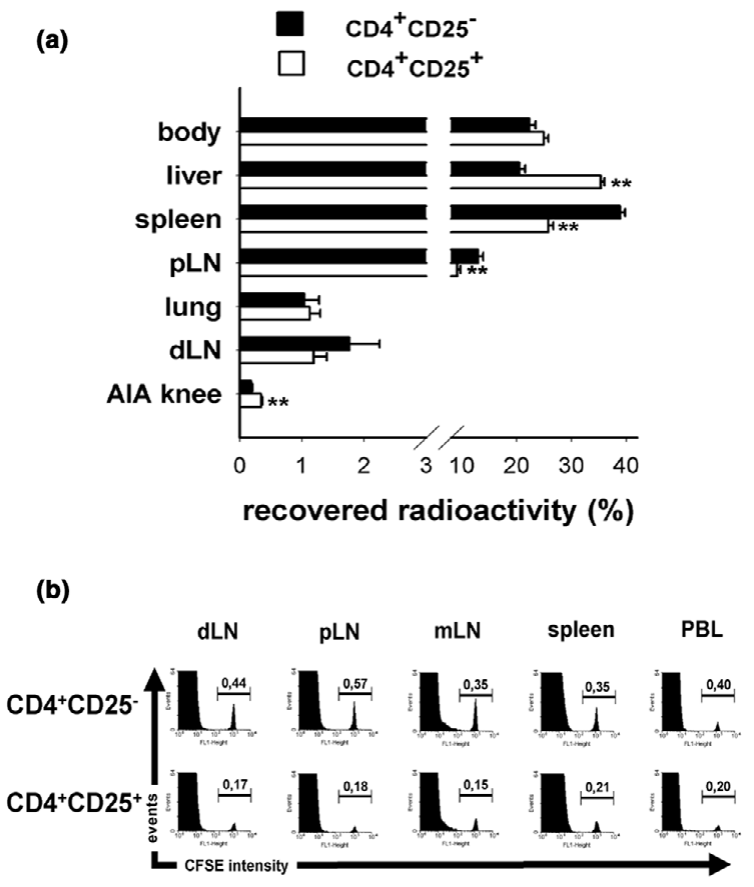
Migration behaviour of regulatory T cells (T_reg _cells). **(a) **CD4^+^CD25^+ ^and CD4^+^CD25^- ^cells were purified by fluorescence-activated cell sorting (FACS) and labelled with ^111^In. Cells (1 × 10^6^) were injected intravenously into antigen-induced arthritis (AIA) mice at day 7. After 24 hours radioactivity in isolated organs and the rest of the body was determined using a γ-counter. Thereafter, the total radioactivity recovered per animal was calculated by adding the counts of the organs and the rest of the body. **(a) **The proportion of radioactivity found in the isolated organs is shown here as a percentage of total recovered radioactivity (*n *= 6; mean ± standard error of the mean; one representative out of two independent experiments; **P < 0.01). **(b) **FACS-purified cells were labelled with 5,6-carboxyfluorescein diacetate succinimidyl ester (CFSE) and injected intravenously. After 24 hours single-cell suspensions from draining lymph node (dLN), nondraining peripheral lymph node (pLN), mesenteric lymph node (mLN), spleen and peripheral blood lymphocytes (PBL) were analyzed by FACS. The percentage of CFSE^+ ^cells of the total CD4^+ ^cells was measured. Histogram plots are gated on CD4^+ ^cells after propidium–iodide exclusion of dead cells (*n *= 3 per group). Higher numbers of CFSE^high ^cells are found in the secondary lymphoid organs in the recipients of CD4^+^CD25^- ^cells.

Compared with CD4^+^CD25^- ^cells, CD4^+^CD25^+ ^T_reg _cells were less abundant in secondary lymphoid organs such as lymph nodes and spleen. Thus, CD4^+^CD25^+ ^cells recirculate through these organs less than do CD4^+^CD25^- ^cells. In the liver, more radioactivity was recovered in recipients of CD4^+^CD25^+ ^cells as compared with CD4^+^CD25^- ^cells. Importantly, CD4^+^CD25^+ ^cells also had a significantly better capacity to enter the inflamed joint than did CD4^+^CD25^- ^cells (Fig. 7a). The level of radioactivity detected in the arthritic joints was low but similar to levels found in transfer experiments with effector T cells [18]. As a control, some mice were injected with CFSE-labelled cells. FACS analysis of the secondary lymphoid organs revealed the presence of viable cells 24 hours after transfer and excluded the possibility that the difference in migration pattern is due to leakage of radioactivity (Fig. 7b). The migration behaviour of CD4^+^CD25^+ ^T_reg _cells does reflect their more activated phenotype, and their ability to enter inflamed joints makes it possible that they act directly at the site of inflammation.

## Discussion

Our findings provide clear evidence that CD4^+^CD25^+ ^T_reg _cells are critical for regulating the severity of AIA in mice. We showed this by manipulating the T_reg _cell numbers using two different approaches: depletion of CD25-expressing cells and transfer of purified CD4^+^CD25^+ ^T_reg _cells. It is important to stress that we depleted CD25-expressing cells in the interval between immunization and AIA induction, because CD25-depletion before immunization profoundly increases the resulting humoral and cellular immune responses [3,12]. These data are consistent with studies conducted in collagen-induced arthritis; however, in these experiments CD25-expressing cells were depleted before immunization with collagen type II, and the resulting more severe arthritis could be interpreted as the result of stronger immunization state [12]. With our experimental design, we were able to examine the effect of T_reg _cells in ongoing joint inflammation directly. Because CD25 is also expressed on activated conventional T cells, it could be assumed that injection of an anti-CD25 antibody would deplete not only T_reg _cells but also effector T cells, but the exacerbated AIA in CD25-depleted mice argues against such a depletion of effector T cells. Accordingly, in control experiments lymph node cells from CD25-depleted mice isolated at the time of induction of AIA were able to mount a similar anti-mBSA response *in vitro *as compared with control mice (data not shown). Furthermore, CD4^+^CD25^+ ^cells isolated from immunized donors can suppress development of AIA (Fig. 4b). Taken together, these data imply that the CD25^+ ^compartment in immunized mice largely consists of T_reg _cells.

AIA induction in CD25-depleted mice resulted in a much more severe arthritis in the acute and chronic stages of disease. We recently showed, with the use of a depleting anti-CD4 antibody, that this acute stage of AIA is already under the control of T cells [15]. Nevertheless, early AIA is dominated by cells of the innate immune system [19], and the exacerbation of arthritis in CD25-depleted mice could be due to a lack of suppression of these cells by T_reg _cells. In accordance with this view, CD4^+^CD25^+ ^T_reg _cells are able to suppress innate immune cells in a model of bacteria-induced colitis [20].

In later stages exacerbated arthritis in CD25-depleted mice is accompanied by increased mBSA-specific proliferation and IgG production. This enhanced responsiveness emerged during arthritis development and is due to sustained T cell activation. Such prolonged T cell activation in the absence of CD4^+^CD25^+ ^cells has also been described in other disease models [21] and is probably the cause of the increased AIA severity. Moreover, the PC61 antibody used in our study has a half-life of approximately 3 weeks *in vivo *(Sutmuller R, personal communication), which makes it possible that T_reg _cell function is not only impaired by depletion but also by blockade of IL-2 binding to CD25 by the PC61 antibody. IL-2 or IL-2 signalling via CD25 has been shown to be critical to the regulatory action of T_reg _cells [22,23]. Also, activation-induced cell death of pathogenic T cells, which is regulated by IL-2, could be impaired by withdrawal of IL-2 signalling and therefore contribute to the observed high levels of cellular immune responses in our study [24].

The fact that depletion of CD4^+^CD25^+ ^T_reg _cells enhances the immune response against the foreign antigen mBSA clearly demonstrates that their suppressive effect is not strictly limited to autoreactive T cells. Taking into consideration that T_reg _cells are also critically involved in the control of immune responses against pathogens [25,26], their physiological function is not just to prevent autoimmunity but also to control the extent of inflammatory reactions in order to prevent tissue damage to the host. Further support for the influence of CD4^+^CD25^+ ^T_reg _cells on arthritis development came from the transfer experiments. When transferred at the time of induction of AIA, CD4^+^CD25^+ ^cells were able to ameliorate ongoing disease. Analysis of the recipients did not reveal a remarkable long-lasting suppression of systemic mBSA-specific immune reactions. Thus, prevention of AIA appears to be possible without inducing anergy or abrogating previously induced T-cell effector functions [27]. In contrast to this, transferred CD4^+^CD25^- ^cells significantly enhance cell-mediated and humoral immune responses.

Furthermore, the homing data presented here demonstrate that CD4^+^CD25^+ ^cells can migrate into the arthritic knee joint. Functional T_reg _cells have repeatedly been found within such effector sites and/or draining lymph nodes, for instance in tolerated allografts [28], in Langerhans islets and pancreatic lymph nodes in inflammation-induced diabetes [29], in chronically inflamed skin in a *Leishmania *infection model [30], and in the mucosa and mesenteric lymph nodes in inflammatory colitis in severe combined immunodeficient (SCID) mice [31]. Interestingly, two recent papers [32,33] reported an accumulation of functional T_reg _cells in the inflamed joints of patients with RA, juvenile arthritis and other rheumatic diseases.

It is most likely that the transferred CD4^+^CD25^+ ^T_reg _cells act in the draining lymph node as well as in the inflamed tissue. Within such a scenario, it could be possible that T_reg _cells inhibit the activation of effector T cells and their subsequent migration to the joints. Such a mechanism was recently speculated in modulation of virally induced immunopathology by T cells [26].

Huehn and colleagues [11] recently demonstrated that CD4^+^CD25^+ ^T_reg _cells can be divided into subsets based on the expression of the integrin α_E_β_7_. Moreover, this marker identifies CD25^- ^T_reg _cells [34]. Both α_E_β_7_-expressing subsets had better capacity to reach the inflamed joint and to prevent arthritis in the AIA model, as compared with α_E_β_7_^- ^T_reg _cells [10]. Thus, suppression at the site of inflammation is also an important part of the activity of T_reg _cells. How this effect is mediated is unclear but an involvement of IL-10 or transforming growth factor-β is possible [20,35,36].

If these hypotheses are correct, then they could explain why the transfer of T_reg _cells after arthritis induction is not effective. On the one hand, transfer of T_reg _cells 24 hours after intra-articular antigen challenge might be too late to inhibit activation of effector T cells and their migration to the joint. Indeed, T-cell activation is an early event in AIA because CD4^+ ^T cell depletion ameliorates the acute stage of the model [15]. On the other hand, it could be possible that the suppressive function of regulatory T cells is switched off under the inflammatory conditions present in the inflamed tissue by factors such as IL-6 or glucocorticoid-induced tumor necrosis factor family-related gene (GITR) and GITR-ligand interactions, abrogating the suppressive effect of T_reg _cells [37]. With this in mind, it could be interesting to investigate whether the accumulated T_reg _cells in patients with arthritis function properly *in vivo *and whether these patients could really benefit from a therapeutic enhancement of T_reg _function, as suggested by some enthusiastic investigators in this field.

In this regard, data on the curative effects of T_reg _cells in experimental disease models are conflicting. To best of our knowledge, a curative effect of CD4^+^CD25^+ ^T_reg _cells has only been demonstrated in the colitis model induced by transfer of CD45RB^high ^T cells into SCID mice [31,38]. In contrast, other authors were unable to demonstrate such an inhibitory effect of T_reg _cells on SCID colitis when they were transferred 1 week after administration of pathogenic CD45RB^high ^T cells [39]. Because arthritis in the AIA model has a hyperacute onset, it could be assumed that the time window for an ameliorative effect of T_reg _cell transfer ends very shortly after intra-articular injection of antigen. However, further studies on the role of T_reg _cells in other arthritis models are clearly needed to clarify whether enhancement in T_reg _cell function might be beneficial in experimental arthritis and perhaps in human disease.

## Conclusion

Our data show that T_reg _cells are critically involved in the control of immune responses that are responsible for the pathogenesis of chronic arthritis. Transfer of such cells can modulate the severity of ongoing inflammatory arthritis but they cannot suppress established disease. Thus, timing of T_reg _cell transfer for therapeutic purposes is of considerable importance.

## Abbreviations

AIA = antigen-induced arthritis; CFSE = 5,6-carboxyfluorescein diacetate succinimidyl ester; DTH = delayed-type hypersensitivity; ELISA = enzyme-linked immunosorbent assay; FACS = fluorescence-activated cell sorting; FCS = fetal calf serum; IFN = interferon; IL = interleukin; mBSA = methylated bovine serum albumin; PBS = phosphate-buffered saline; RA = rheumatoid arthritis; SCID = severe combined immunodeficient; T_reg _= regulatory T cell.

## Competing interests

The author(s) declare that they have no competing interests.

## Authors' contributions

OF purified the anti-CD25 hybridoma and purified the monoclonal antibodies from the supernatant; planned and conducted all animal experiments, including ELISA and ELISPOT analysis; and drafted the manuscript. PKP and MG scored the histological changes in arthritic joints. KS, JH and AH conducted the migration experiments, as well as the α_E_β_7 _transfer experiments. RB supervised the project and participated together with AS and AR in the design of the study and its coordination, and helped to draft the manuscript. All authors read and approved the final manuscript.
